# Aurora A’s Functions During Mitotic Exit: The Guess Who Game

**DOI:** 10.3389/fonc.2015.00290

**Published:** 2015-12-21

**Authors:** David Reboutier, Christelle Benaud, Claude Prigent

**Affiliations:** ^1^Unité Mixte de Recherche 6290, Équipe labellisée Ligue, Centre National de la Recherche Scientifique, Rennes, France; ^2^Institut de Génétique et Développement de Rennes, Université Rennes 1, Rennes, France

**Keywords:** mitosis, cytokinesis, Aurora kinase, central spindle, cancer, microtubule

## Abstract

Until recently, the knowledge of Aurora A kinase functions during mitosis was limited to pre-metaphase events, particularly centrosome maturation, G2/M transition, and mitotic spindle assembly. However, an involvement of Aurora A in post-metaphase events was also suspected, but not clearly demonstrated due to the technical difficulty to perform the appropriate experiments. Recent developments of both an analog-specific version of Aurora A and small molecule inhibitors have led to the first demonstration that Aurora A is required for the early steps of cytokinesis. As in pre-metaphase, Aurora A plays diverse functions during anaphase, essentially participating in astral microtubules dynamics and central spindle assembly and functioning. The present review describes the experimental systems used to decipher new functions of Aurora A during late mitosis and situate these functions into the context of cytokinesis mechanisms.

## Introduction

Aurora A and Aurora B are two major serine/threonine kinases participating in mitosis regulation. From an evolutionary point of view, equatorial Aurora B kinase likely appeared before polar Aurora A kinase (1). Although the latest is a derivative of Aurora B, it possesses its own expression pattern and its own crucial mitotic functions. Aurora A was discovered in the 90s by Glover and colleagues in a screen designed to identify genes that affect centrosomes cycle in *Drosophila* (2). Since this first study, Aurora A has been the focus of many attentions in fundamental and medical research, because the loss of control of its expression or activity has been directly linked to cancer. Several functions of Aurora A kinase during mitosis have been well established. Aurora A regulates mitotic entry through phosphorylation of CDC25B phosphatase (3) or PLK1 kinase (4, 5). Aurora A also contributes to DNA damage (6) and to spindle assembly checkpoints (SAC) (7). Once the cell is engaged into mitosis, Aurora A participates in mitotic spindle assembly and functioning. Aurora A triggers centrosome maturation by recruiting NDLE1 (8) and TACC3 (9). In prometaphase, Aurora A participates in the regulation of microtubule dynamics and contributes to the recruitment of factors involved in the dynamic instability of microtubules, including DDA3 (10), MCAK (11), ch-TOG (12, 13), and KIF2A (14). Aurora A is also involved in the recruitment of proteins that move along microtubules, for example, Kinesin 5 (Kif11) (15) and p150Glued (16). Lastly, Aurora A has been shown to be involved in chromatin driven microtubules nucleation through NEDD1 phosphorylation (17). These functions of the kinase are closely related to its localization. Indeed, Aurora A is located to centrosomes in G2 and both to centrosomes and to mitotic spindle poles during mitotic spindle assembly. Interestingly, the kinase is also found associated with the central spindle and later on the midbody during mitotic exit. In spite of the description of these late mitotic localizations, there was no formal data demonstrating the involvement of Aurora A into mitotic exit until recently, mainly because of technical limitations. Indeed, studies investigating the functions of Aurora A have involved modifying Aurora A activity by RNA interference depletion of the protein (siRNA), by over expression (18, 19) and/or by the use of mutants (active, inactive, hyperactive, or non-degradable) (7, 20–24). The major outcome of such experiments is the failure of centrosome maturation (23). During G2, the cell prepares to enter mitosis and numerous proteins required for microtubule nucleation are recruited to centrosomes to participate in the mitotic spindle assembly. Defects in centrosome maturation frequently result in a longer G2/M transition and perturb the mitotic spindle assembly, thus maintaining the SAC active. The active SAC prevents the metaphase/anaphase transition, thereby most of the time impedes the investigation of Aurora A functions beyond this step. In view of the crucial role of Aurora A in spindle organization before anaphase and its post-metaphase localization, an implication of Aurora A in the regulation of the spindle during mitotic exit would not be surprising. In order to better understand late mitotic events, potential late mitotic functions of Aurora A should be investigated. Indeed, in the early 2000s, studies that had resulted in only partial perturbation of the activity of the kinase have pointed out some late mitotic functions for Aurora A. The specific involvement of Aurora A during mitotic exit was confirmed only recently by the use of pharmacological inhibition of the kinase. The present review focuses on the experimental systems that have been used to decipher late mitotic functions of Aurora A and discusses these functions in the context of mitotic exit.

## The First Clues of the Late Mitotic Involvement of Aurora A

### Targeting of the Kinase by Cellular Microinjection of Anti-Aurora A Antibodies

The first study that brought some insight in the involvement of Aurora A in mitotic exit was led by Marumoto and colleagues (24). The aim of this study was to understand the physiological functions of human Aurora A. In this context, the authors first depleted the kinase by siRNA in HeLa cells. In cells reaching the best depletion efficiency, they observed a classical absence of mitotic entry. Yet, when only partial depletion was achieved, they observed chromosomes misalignment and some cells presented multiple nuclei that are often synonymous of cytokinesis failure. To pin point the specific role of Aurora A throughout the different phases of mitosis, the authors inhibited the kinase by microinjection of affinity purified anti-Aurora A polyclonal antibodies at different time of mitosis progression. Injection of HeLa cells with the antibodies in late G2 triggered a delay in mitotic entry, a prolonged duration of early (prometaphase and metaphase) and late mitosis (anaphase and telophase), a defect in chromosomes congression, the appearance of mitotic spindle with multiple spindle poles, and an unequal chromosomes segregation. All these phenotypes have now commonly been described as typical of the inhibition of Aurora A. Instead, microinjection of anti-Aurora A antibodies after centrosomes separation and chromosomes alignment onto metaphase plate triggered a cytokinesis defect. Albeit sister chromatids separated and the cleavage furrow formed, meaning that the acto-myosin ring assembled and could contract, cytokinesis aborted, and daughter cells fused. These data, which strongly suggested for the first time that the Aurora A kinase could be involved in mitotic exit, were reinforced by the first demonstration that Aurora A was not only localized on the centrosomes and the mitotic spindle poles but also on the central spindle and the midbody. This pioneer study was particularly interesting since it was the first time that Aurora A was inhibited in a precise window of time, which specifically targeted mitotic exit. Yet, effects of antibodies microinjections are difficult to interpret because there is no real negative control that assesses putative off target effects. Moreover, since the antibodies used to inhibit Aurora A were obtained after injection of the regulatory domain of the kinase into a rabbit (amino acids 1–129), the catalytic domain of the kinase is most likely not targeted. As the authors did not test the effect of this polyclonal antibody onto Aurora A’s catalytic activity, the real effect of the injection into culture cells is thus difficult to assess and cellular data have to be examined cautiously.

### Indirect Stabilization of the Kinase

More recently, a second study brought interesting data concerning putative late mitotic functions of Aurora A through indirect action on the stability of the kinase. In HeLa cells, Floyd and colleagues explored the time course of APC/C^Cdh1^ activity and functioning (25). As previously described in *Xenopus* cell free extracts (26, 27), authors observed that it was involved in Aurora A kinases degradation. Indeed, they found that siRNA-mediated Cdh1 depletion led to a stabilization of Aurora A and B that were not degraded anymore during mitotic exit. In parallel, Floyd and colleagues examined whether APC/C^Cdh1^ could be involved into mitotic exit through time-lapse recordings of Cdh1-depleted cells. They measured the time taken from anaphase onset to cleavage furrow ingression completion and found that, after knock-down of Cdh1, this time was reduced. Authors also observed that sister chromatids segregation occurred more rapidly, likely indicating that microtubules dynamics was modified. This was indeed the case, since in Cdh1-depleted cells, the robustness of the central spindle was weaker, and there was an exaggerated growth of astral microtubules at the spindle poles, which persisted abnormally during telophase and abscission. To test whether the Aurora kinases were responsible for such phenotypes, the authors then expressed non-degradable forms of Aurora A or B. They found that expression of each one could mimic the depletion of Cdh1. Interestingly enough, the authors also remarked that overexpression of a non-degradable version of Aurora B reduced the degradation of Aurora A. As Aurora A appears to be degraded earlier than B, the authors suggested that destruction of Aurora A in anaphase may be sufficient to prevent proper anaphase spindle organization, and Aurora A is the likely critical target of APC/C^Cdh1^ at anaphase onset. To confirm this hypothesis, Floyd and colleagues then tested whether depletion of Aurora A by siRNA could rescue Cdh1 depletion. In agreement with previously published data, cells depleted for Aurora A were delayed in prometaphase and when they entered into metaphase, they frequently presented fragmented poles. When Cdh1 and Aurora A were depleted simultaneously, the over elongation of anaphase spindle observed when Cdh1 was depleted alone was partially reduced, thus confirming a role played by Aurora A in central spindle dynamics.

To further investigate the mechanisms that could be responsible for spindle over elongation, Floyd and colleagues analyzed the distribution of Aurora kinases in Cdh1-depleted cells. The authors first observed a persistence of the Aurora A staining at spindle poles from anaphase to G1 stage. This persistence of Aurora A correlated with the increase of astral microtubules density indicating that the degradation of Aurora A at this stage of the mitosis might be used to regulate astral microtubules dynamics. Concomitantly with the polar stabilization of the kinase, Cdh1 depletion also triggered the polar retention of TPX2, which is a well-characterized Aurora A activator involved into mitotic spindle microtubules nucleation in a Ran-GTP-dependent manner. The staining of EB1 protein, which localized at the plus tip of growing microtubules, was not modified, indicating that Cdh1 likely regulates global microtubules stability rather than microtubules growth. In parallel to these results, the authors also observed that Cdh1 depletion altered not only the stability but also the distribution of Aurora B. Instead of being localized to the central spindle midzone, authors found that the kinase was weakly localized to the midzone and rather accumulated to a diffuse band in the region of the equatorial cortex. Mklp2, which mediates the localization of Aurora B to the central spindle midzone, was also relocalized to the equatorial cortex. In contrast to MKLP1, a component of the Centralspindlin complex, PRC1 and PLK1, was correctly localized. The authors proposed that alteration of the central spindle density and structure could be due to the weak localization of MKLP2 and Aurora B to the midzone, but MKLP1, PRC1, and PLK1 are sufficient to drive the assembly of a weak spindle midzone that allows the initiation of the cleavage furrow.

Altogether, these results suggest a predominant role of Aurora A in the regulation of early anaphase spindle dynamics, notably in the stabilization of astral microtubules, whereas Aurora B would be involved later, likely in central spindle stability. However, in this experimental system, a function for Aurora A in central spindle assembly cannot be definitively ruled out since it may be hidden by the phenotype triggered by the mislocalization of Aurora B. Another possibility could be that Aurora A and B share common substrates and could participate in the same pathways during mitotic exit.

### Conditional Knock-Out of the Kinase

Work by Hégarat and colleagues (28) has pursued on the notion of cooperation between Aurora A and B, a few years later. In their paper, the authors explored Aurora A’s functions through conditional knock-out of the protein. They took advantage of the DT40 chicken cells to set up a system in which the two WT alleles of Aurora A were disrupted. This system was chosen to ensure complete Aurora A depletion and avoid the potential side effects triggered by kinase inactivation and protein removal. Using this strategy, the authors first confirmed previous results: they observed mitotic cells with unaligned chromosomes, mitotic spindle with reduced volumes, and defective PLK1 activation in G2 phase. Interestingly, the simultaneous impairment of Aurora A expression and chemical inhibition of Aurora B (with 60 nM AZD1152, a potent Aurora B inhibitor) triggered a complete absence of chromosomes segregation followed by their decondensation. This defect was accompanied by the persistence of long and stable MT fibers in Aurora A^KO^/Aurora B inhibited cells, whereas Aurora B inhibited cells presented the classical spindle contraction typical of anaphase onset. In Aurora A^KO^ cells, astral microtubules appeared partially stable, but chromosomes finally separated. Curiously, the authors did not mention any further effect in later phases of mitosis. Altogether, these results suggested a collaborative role for Aurora A and Aurora B in chromosomes segregation during early anaphase, through control of mitotic spindle microtubules stability. This observation could be the result of substrate or pathway redundancy and point to the complex interplay between centrosomal and centromeric functions in regulating mitotic spindle dynamics [for further information, see the review by Hochegger and colleagues (1)]. Even though the experimental system used by Hégarat and colleagues allowed a real-specific targeting of Aurora A, it did not allow the inhibition of the kinase within an accurate window of time. Consequently, many events that require the presence of Aurora A or its activity remained inaccessible. This drawback was solved 2 years later through pharmacological inhibition of the kinase.

## The Validation of Aurora A’s Late Mitotic Involvement through Pharmacological Inhibition

### The Chemical Genetics Strategy

The best way to address the late mitotic functions of Aurora A is to pharmacologically target the kinase just after the metaphase to anaphase transition, once the SAC is satisfied. Our group was the first who succeeded in developing such an approach (29). To perform this task, we used chemical genetics techniques that consist in modifying the catalytic domain of the kinase to make it sensitive to an ATP analog that has no effect on the WT Aurora A kinase. This system thus, in addition to allow the timely control of Aurora inhibition, enables us to detect any off-target effects of the ATP analog by using the WT kinase as a negative control. To generate an Aurora A variant with an enhanced sensitivity to ATP analogs, we have modified the specificity of the ATP-binding pocket of the kinase by converting leucine 210 into an alanine [L210A Aurora A mutant referred to hereafter as analog-sensitive Aurora A (as-AurA)]. *In vitro*, recombinant as-AurA was as active as the WT version of the kinase (wt-AurA) but was specifically inhibited by the ATP analog 1-Na-PP1 that had no effect on wt-AurA. We generated stable U2OS human cell lines, expressing RNA interference resistant GFP-tagged versions of wt-AurA or as-AurA alleles under the Aurora A minimal promoter (30). In these cells, as-AurA localized similarly to what has been previously described for WT Aurora A and was able to rescue endogenous Aurora A depletion, indicating it is fully functional. Treatment of cells only expressing wt- or as-AurA with 1-Na-PP1 for 24 h, substantially increased the percentage of multipolar or fragmented spindle poles in as-AurA expressing cells [as previously described by Asteriti and colleagues (12)], whereas it had no effect in wt-AurA expressing cells. Altogether, these data show that our chemical genetics system is valid.

To study the effect of Aurora A inhibition just after the metaphase to anaphase transition, we applied 1-Na-PP1 in a timely fashion on wt- or as-AurA cells. When Aurora A was inhibited in metaphase, most cells were blocked and the mitotic spindle collapsed with the two spindle poles closely juxtaposed to the chromatin. When Aurora A was inhibited within the first few seconds of anaphase, the chromosomes separated, but rapidly stopped and cells did not undergo telophase or cytokinesis, leading to the generation of binucleated cells. In these cells, the central spindle was largely disorganized or even absent, leading to the absence of anaphase B. Clearly, these results indicate that Aurora A is both involved in mitotic spindle stabilization during metaphase and later in central spindle assembly during anaphase.

We then searched to identify the defective molecular mechanism leading to anaphase spindle abortion. Central spindle assembly is a complex process involving diverse molecules with highly specific functions. The evolutionarily conserved Centralspindlin complex is a major player in this process. Appropriate localization of Centralspindlin in *Drosophila* depends on the dynactin complex and depletion of the dynactin subunit p150Glued in *Drosophila* S2 cells perturbs Pav-KLP (the ortholog of MKLP1) localization and central spindle organization (31). Our data showed that inhibition of Aurora A during early anaphase-triggered mislocalization of MKLP1 and the accumulation of p150Glued at mitotic spindle poles. Furthermore, we investigated the molecular mechanism involving p150Glued and found that it was phosphorylated by Aurora A on serine 19. This residue belongs to the microtubule-binding domain of p150Glued that is known, in *Drosophila*, to be phosphorylated by Aurora A in pre-anaphase stages (16). Moreover, in interphasic human cells, p150Glued phosphorylation by the PKA kinase has previously been shown to regulate its affinity for microtubules (32). Interestingly, the mutation of serine 19 into an alanine (S19A, which is non-phosphorylable) mimics the inhibition of Aurora A, whereas the mutation into aspartic acid (S19D, that mimics a constitutive phosphorylation) partially rescues Aurora A inhibition.

Currently, the exact mechanism involving Aurora A and p150Glued in central spindle assembly remains to be deciphered. The p150Glued protein can interact with EB1, a microtubule-associated protein involved in microtubule nucleation (33, 34). This interaction, between p150Glued and EB1, is necessary for microtubule binding to centrosomes (33) and for microtubule nucleation (35–37). The C-terminus of EB1 binds to the N-terminus of p150Glued, and this event decreases microtubule shortening and increases rescue frequency and the growth rate of microtubules, thereby favoring microtubule elongation (33, 36). Aurora A depletion results in the disconnection of centrosomes from mitotic spindle poles in *Drosophila* (16), and inhibition of Aurora A seems to be involved in central spindle microtubule nucleation (29). Both of these effects resemble those of EB1 inactivation (33, 35–37). Consequently, phosphorylation of p150Glued serine 19 by Aurora A could be involved in central spindle assembly through an EB1 function. Another hypothesis involves Kinesin 5. Uteng and colleagues have shown that the dynein/dynactin complex is responsible for the transport of the kinesin 5 motor toward the poles (38). As kinesin 5 is required for accurate central spindle assembly (39–42), a defect in p150glued localization during early anaphase could trigger Kinesin 5 mislocalization and concomitant defects in central spindle assembly.

### Targeting of Aurora A by a Small Molecule Inhibitor

During the same period, Lioutas and Vernos also demonstrated the involvement of Aurora A in central spindle assembly by using the small molecule inhibitor MLN8237 (43). MLN8237 is a selective Aurora A inhibitor that has >200-fold higher selectivity for Aurora A than Aurora B in cell free assay. In HeLa cells, the authors determined that 250 nM MLN8237 was the concentration that gave the best inhibitory effect on Aurora A without any effect on Aurora B. Similarly to our results, when cells were treated with MLN8237 during metaphase, mitotic spindle collapsed with both centrosomes traveling toward each other, confirming that Aurora A activity is required for mitotic spindle stability. When cells were treated with MLN8237 at anaphase onset, they progressed through anaphase until cytokinesis but with a slower kinetics than control cells. Moreover, Aurora A inhibited cells presented several chromosome segregation defects, including chromatin bridges and lagging pieces of chromosomes. As Aurora A is an important regulator of microtubule stability during mitotic spindle assembly, the authors examined whether the microtubule function was compromised. The pole-to-pole distance during chromosomes segregation was strongly reduced, due to a decrease in central spindle elongation. Additionally, central spindle appeared weaker and more disorganized than in control, while kinetochore fibers appeared to shorten slightly faster than in control cells. Overall, these data indicated that Aurora A activity is involved in chromosomes segregation and is strongly required for central spindle microtubules assembly and organization during mitotic exit.

TACC3 is a substrate of Aurora A that is involved in microtubules stabilization in partnership with chTOG/XMAP215. It is phosphorylated by the kinase on serine 558 during mitotic spindle assembly (9, 44). Immunofluorescence experiments showed that phosphorylated TACC3 is localized on mitotic spindle poles in pre-anaphase and on the poles and the central spindle during mitotic exit. Moreover, the phospho-TACC3 signal was strongly reduced when cells were treated with MLN8237. Depletion of TACC3 by siRNA-triggered effect similar to those induced by Aurora A inhibition: the progression of cells through mitotic exit was slower, the elongation of central spindle was decreased, and the microtubule fluorescence intensity of central spindle was reduced when compared to control. Interestingly, while the exogenous expression of the WT version of TACC3 partially rescued the depletion of TACC3, it was not the case for the non-phosphorylable S558A mutant version. The authors finally realized depolymerization and regrowth assays to further characterize the role of Aurora A and TACC3 into the regulation of central spindle dynamics. When cells were incubated on ice, depolymerization occurred faster in MLN8237-treated cells or in TACC3-depleted cells than in control. Interestingly, for TACC3-depleted cells treated with MLN8237, depolymerization was not enhanced when compared with TACC3-depleted cells alone, likely meaning that Aurora A and TACC3 act in a similar pathway. Similarly, whereas in control cells microtubule regrowth was very efficient, in MLN8237-treated or TACC3-depleted cells, microtubule regrowth was strongly delayed. Altogether, these data strongly confirm the late mitotic involvement of Aurora A by using a small molecule pharmacological inhibition of the kinase.

## Conclusion

Historically, mitotic spindle assembly is considered as a critical event for proper chromosome segregation and mitosis progression. Nonetheless, cytokinesis is also emerging as a crucial event of cell division. Even though a dividing cell manages to correctly build its metaphase spindle, a subsequent cytokinesis failure would also lead to polyploidy or aneuploidy and cause genome instability. Whereas the pre-anaphase functions of the Aurora A kinase are extensively documented, studies deciphering the cytokinetic functions of Aurora A remained limited until recently. The various works presented in this review confirm that Aurora A cytokinetic functions are not anecdotal, and understanding these functions is of critical importance for the comprehension of cytokinesis.

**Figure 1:**
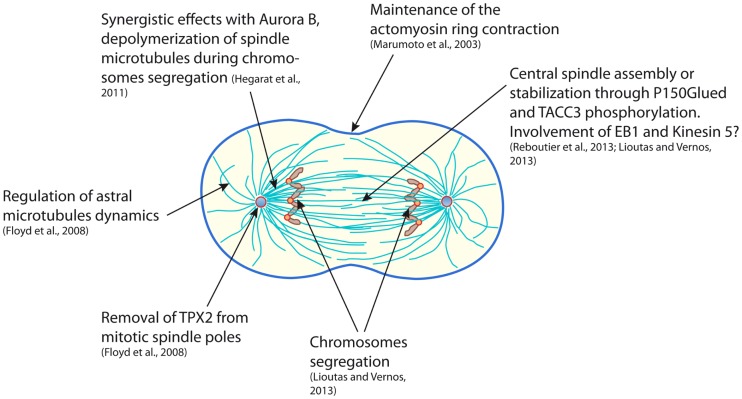
**Scheme representing a mammalian cell in early cytokinesis and summarizing the functions of the Aurora A kinase during mitotic exit**.

Despite the highly different approaches that were used in the works presented here, Aurora A clearly appears to be directly involved in astral microtubules stability and central spindle robustness, both being determinant for an accurate cytokinesis (Figure 1). The few discrepancies observed in the different studies may mainly reflect the heterogeneous means that were used to target Aurora A: partial or total depletion, indirect stabilization or pharmacological inhibition of the kinase. Moreover, Aurora A has been shown to also perform functions that are independent of its kinase catalytic activity, thus carrying out a depletion or an inhibition of Aurora A may target different function of the kinase and result in a different outcome (45, 46). According to the studies described in the present review, Aurora A likely exerts many functions from the “dawn to the dusk” of cytokinesis. Some of the events participating in cytokinesis are very dynamic and last only few minutes (for example, central spindle assembly). Under these circumstances, *in vitro* experimental systems could in the future be highly valuable, notably “artificial centrosomes” that are constituted of Aurora A coated beads nucleating aster-like structures in *Xenopus* egg extract (47). This system enables to reassemble an anaphase spindle showing interesting features in terms of size, shape, and biochemistry (48). However, the highly dynamic nature of the remodeling of the mitotic spindle also calls for live cell video-microscopy approaches in order to decipher Aurora A’s late mitotic functions. Even though the chemical genetics system that we have developed was up to date and the only way to evaluate the effect of a real-specific inhibition of the kinase, the emergence of small molecule inhibitors that appear more and more specific should soon open the way to extensive study of the hidden functions of Aurora A kinase.

Its function in pre-anaphase stages of mitosis has made Aurora A as a potentially interesting target for cancer therapy and has led to the development of Aurora A-specific pharmacological inhibitors. The Aurora A inhibitor, MLN8237 (also known as Alisertib), is now in clinical phase III study (49). Paradoxically, the understanding of Aurora A’s functions, during interphase, asymmetric division or mitotic exit, is at its dawn. Moreover, we now know that both a gain and a loss of activity of Aurora A can lead to carcinogenesis, depending on the mode of cell division (50–53). The fact that Aurora A appears more and more as a pleitropic protein should thus lead to consider cautiously the opportunity to inhibit its activity to treat cancer.

## Author Contributions

DR conceived the structure of the review, critically analyzed the literature, wrote the manuscript, and prepared figures. CB discussed extensively review structure and contents, contributed to manuscript writing. CP critically analyzed the literature, contributed to manuscript writing.

## Conflict of Interest Statement

The authors declare that the research was conducted in the absence of any commercial or financial relationships that could be construed as a potential conflict of interest.
